# Mesenchymal stem cells expressing interleukin-18 suppress breast cancer cells *in vitro*

**DOI:** 10.3892/etm.2015.2286

**Published:** 2015-02-11

**Authors:** XIAOYI LIU, JIANXIA HU, SUYUAN SUN, FUNIAN LI, WEIHONG CAO, YU WANG, ZHONGLIANG MA, ZHIGANG YU

**Affiliations:** 1 Department of Galactophore, The Second Hospital of Shandong University, Jinan, Shandong 250033, P.R. China; 2 Department of Galactophore, The Affiliated Hospital of Qingdao University, Qingdao, Shandong 266003, P.R. China; 3 Stem Cell Research Center, The Affiliated Hospital of Qingdao University, Qingdao, Shandong 266003, P.R. China

**Keywords:** breast cancer, cancer gene therapy, mesenchymal stem cell, interleukin-18, gene transduction

## Abstract

Breast cancer is the most common malignancy among females throughout the world. Current treatments have unsatisfactory outcomes due to the dispersed nature of certain types of the disease. The development of a more effective therapy for breast cancer has long been one of the most elusive goals of cancer gene therapy. In the present study, human mesenchymal stem cells derived from umbilical cord (hUMSCs) genetically modified with interleukin 18 (IL-18) gene were used to study the effect of hUMSCs/IL-18 on the growth, migration and invasion of MCF-7 and HCC1937 cells *in vitro*. The hUMSCs could be efficiently modified by lentiviral systems and stably expressed IL-18 protein. hUMSCs/IL-18, but not hUMSCs without the IL-18 gene transduction, significantly suppressed the proliferation, migration and invasion of the MCF-7 and HCC1937 cells. The mechanism of this proliferation suppression may have involved the induction of G_1_- to S-phase arrest of the breast cancer cells by the hUMSCs/IL-18. In conclusion, hUMSCs/IL-18 can suppress the proliferation, migration and invasion of breast cancer cells *in vitro* and may provide an approach for a novel antitumor therapy in breast cancer.

## Introduction

Breast cancer is the most common malignancy among females throughout the world. Combined therapies of surgery, chemotherapy, radiotherapy, endocrine therapy and targeted therapy are important tools in the treatment of breast cancer; however, current treatments can lead to a number of unsatisfactory outcomes, predominantly due to the difficulty accessing certain tumor sites, the dispersed nature of the disease and the toxicity of the treatments. The development of a more effective therapy for breast cancer has long been one of the most elusive goals of cancer gene therapy (1).

Recently, there an been an increased focus on the potential of mesenchymal stem cells (MSCs) for use in cell therapy and tissue engineering due to their multipotency and ability to secrete growth factors (2,3). Furthermore, as a result of their characteristics of hypo-immunogenicity and immunomodulation, MSCs have been shown to be effective as gene delivery vehicles for therapeutic purposes (4–6). It has also been demonstrated that MSCs can migrate towards tumors in response to chemokines produced by tumor cells, when introduced into the organism by systemic intravenous administration (7,8), and contribute to the population of supportive stromal cells around tumors (9,10). These observations support the development of therapeutic strategies based on the local production of tumoricidal biological agents by gene-manipulated MSCs.

Lentiviruses exhibit a number of desirable qualities as vehicles for gene delivery for use in experimental studies and gene therapy, as they can transfect dividing and non-dividing cells, show stable transgene expression and have a low toxicity and immunity and a large cloning capacity (9 kb) (11,12). Interleukin-18 (IL-18), a cytokine that was formally known as interferon-γ (IFN-γ)-inducing factor, exhibits structural and functional similarities to IL-1. IL-18 is involved in numerous biological activities, acting through its ability to stimulate innate immunity and T-helper type 1 (Th1)- and Th2-mediated responses (13,14). Furthermore, IL-18 can exert an antitumor effect by enhancing the activity of natural killer (NK) cells, reducing tumorigenesis, inducing tumor cell apoptosis and inhibiting tumor angiogenesis (15). Contradictorily, recent studies have shown that, in addition to the antitumor activity of IL-18 in the immune system, IL-18 can exert pro-cancer effects when produced by cancer cells, acting to promote cell proliferation and migration. The autocrine action of IL-18 can, for example, induce cell migration in gastric cancer and melanoma (16,17). In addition, clinical studies have associated this marker in the serum with the prognosis of bladder cancer, glioma and breast cancer, particularly metastatic breast cancer (13,18,19). Srabović *et al* (20) found that patients with breast cancer who exhibited liver and bone metastasis had significantly increased serum IL-18 levels relative to healthy females. Taking into account the involvement of IL-18 in breast cancer progression and metastasis, Yao *et al* (21) suggested that IL-18 could play dual functions in drug resistance and tumor metastasis. Several studies have found that dendritic cells (DCs) loaded with lysed tumor cells and IL-18 could stimulate Th1 responses against glioma antigens and markedly enhance the cytotoxic efficacy of cytotoxic T lymphocytes towards tumor cells (19,22); however, few data exist regarding the effect of MSCs modified with IL-18 gene in human tumors, and there is an urgent requirement for their effect on different types of tumors to be studied. The objective of the present study, therefore, was to transduce human MSCs from umbilical cord (hUMSCs) with Lenti-IL-18 recombinant virus and observe the antitumor effect, in order to determine whether hUMSCs modified with IL-18 gene could suppress the proliferation, invasion and migration of breast cancer cells *in vitro*.

## Materials and methods

### MSC preparation

Umbilical cord was obtained from a healthy 25-year-old female who had given birth to a healthy, term fetus. The female had no family history of genetic disorders or cancer and had tested negative for hepatitis B, hepatitis C, human immunodeficiency and Epstein-Barr viruses, as well as cytomegalovirus and syphilis. The umbilical cord collection was approved by the Institutional Medical Research Ethics Committee of the Qingdao maternity hospital (Qingdao, China). Fully informed consent was obtained from the pregnant female several weeks prior to delivery.

The preparation of the hUMSCs was performed in the laminar flow laboratory. Briefly, the umbilical cord was washed twice with phosphate-buffered saline (PBS) and cut with scissors into pieces measuring 1–2 mm^3^ in volume. These tissue pieces were plated in a cell culture dish (Corning^®^ 430597; Corning, Inc., Palo Alto, CA, USA) in low-glucose Dulbecco’s modified Eagle’s medium (DMEM; HyClone, GE Healthcare Life Sciences, South Logan, UT, USA) supplemented with 10% fetal bovine serum (FBS; HyClone, GE Healthcare Life Sciences). The cell cultures were maintained at 37°C in a humidified atmosphere with 5% CO_2_. Following three days of culture, the medium was replaced in order to remove the tissue and non-adherent cells; medium replacement was performed twice weekly thereafter. Subsequent to the cells reaching 80% confluence, 0.125% trypsin was used to detach the adherent cells (passage 0), which were then passaged in the cell culture dish. The MSC culture and expansion was performed in a laminar flow laboratory for four passages to prepare the final, sterile cell products. The cells were subsequently stained with a double label and analyzed by flow cytometry using a FACSCalibur™ flow cytometry system (Becton Dickinson, Franklin Lakes, NJ, USA).

### Transduction of hUMSCs

A lentivirus construct containing the green fluorescent protein (GFP) and human IL-18 genes or blank lentivirus vector was used (GenePharma, Beijing, China). The hUMSCs were plated at a density of 10^4^ cells per well in a 96-well plate (Corning 3896; Corning, Inc.). After 24 h, the viruses were added to the medium to infect the MSCs at 10, 30, 50, 70 and 100 pfu/cell (in order to explore the optimal dose), and the plates were then centrifuged at 600 × g for 90 min at 37°C prior to culture overnight. Following culture, the cells were washed with PBS, the medium was replaced with a fresh culture medium and the cells were further incubated for up to 72 h. hUMSCs transfected with blank vector were used as controls. Transduction efficiency was determined using an inverted fluorescence microscope based on expression of the GFP gene. The percentage of cells positive for GFP was determined using flow cytometry by setting a gate according to the control; 10,000 cells were evaluated in each experiment. Effective transduction was confirmed by IL-18 enzyme-linked immunosorbent assay (ELISA) of the culture supernatant (KB1138; Shanghai Kaibo Biochemical Reagent Co., Ltd., Shanghai, China) at 24, 48, 72 h and one week after transduction.

### Semi-quantitative reverse transcription-polymerase chain reaction (RT-PCR)

Extraction of total RNA from the cultured cells was performed using TRIzol^®^ reagent (Invitrogen™, Life Technologies, Carlsbad, CA, USA) in accordance with the manufacturer’s instructions. An ABI Prism^®^ 7500 Sequence Detection System (Applied Biosystems, Foster City, CA, USA) with SYBR^®^ Green I dye (Molecular Probes^®^, Life Technologies) was used for cDNA amplification and quantification. The primer sequences for IL-18, which were designed using Primer Express v2.0 software (Applied Biosystems), were as follows: forward: 5′-GAATAAAGATGGCTGCTGAACC-3′ and reverse: 5′-CCTGGGACACTTCTCTGAAA-3′. The reference gene GAPDH (146 bp) was used as a control. The reaction conditions were as follows: Initial incubation at 94°C for 5 min followed by 35 cycles of incubation at 98°C for 10 sec, 57°C for 30 sec and 72°C for 30 sec, with a final extension step at 72°C for 10 min. The PCR products were subsequently analyzed by electrophoresis and separated on the 2.0% agarose gel. The expression of the IL-18 gene was normalized to the geometric mean of the expression of the reference gene, GAPDH, in order to control the variability in expression levels. Normalized expression was calculated using the following formula: 2^−[(Ct of IL-18) − (Ct of GAPDH)]^, where Ct represents the threshold cycle for each transcript. The results were expressed as the average density of the positive bands obtained from three independent experiments.

### Western blot analysis

The cells were washed with ice-cold PBS and lysed with M-PER^®^ Mammalian Protein Extraction Reagent (78501; Pierce Biotechnology, Thermo Scientific, Inc., South Logan, USA). Sodium dodecyl sulfate-polyacrylamide gel electrophoresis was used to separate equal quantities of proteins, which were subsequently transferred to polyvinylidene fluoride membranes (IPVH00010 Immobilon-P; Merck-Millipore, Darmstadt, Germany). The membranes were probed with antibody against IL-18 (rabbit anti human; 1:1,000; #ab68435; Abcam Trading (Shanghai) Company Ltd., Shanghai, China). Horseradish peroxidase (HRP)-conjugated anti-rabbit secondary antibody (Cell Signaling Technology, Inc., Beverly, MA, USA) was used as a probe, and the immunoreactive bands were visualized with the Immobilon Western Chemiluminescent HRP Substrate (Merck-Millipore). The intensity of the bands was measured using ImageJ software (National Institutes of Health, Bethesda, MD, USA).

### ELISA

Culture supernatant was collected at different time-points, and the level of IL-18 was quantified using an ELISA kit (KB1138; Kangbo Co.) in accordance with the manufacturer’s instructions. The absorbance was read at 450 nm. The minimum sensitivity of detection was 1.0 pg/ml.

### Direct coculture of MCF-7 and HCC1937 cells with hUMSCs/IL-18 in vitro

The MCF-7 and HCC1937 cells (donated by the Central Laboratory of the Affiliated Hospital of Qingdao University, Qingdao, China) were respectively seeded in the bottom chamber of a 24-well Transwell^®^ culture system (Corning, Inc.) containing 600 μl medium (low-glucose DMEM supplemented with 10% FBS). The hUMSCs/IL-18 were dispersed onto the inserts of the Transwell dishes with 0.4-μm pore size.

Four groups were included in the experiment: Tumor cells (MCF-7 or HCC1937), hUMSCs + tumor cells, hUMSCs/vector + tumor cells and hUMSCs/IL-18 + tumor cells. At least five wells were randomly examined each time, and all experiments were repeated twice.

### Tumor cell proliferation assays

After one, three and five days of coculture, triplicates of hUMSCs and tumor cells (1×10^4^ cells per well) in each group were respectively plated into 96-well plates and incubated for 24 h at 37°C. A total of 10 μl cell counting kit 8 (CCK-8) solution was then added into the culture medium for incubation for 3 h at 37°C. Following incubation, the plates were subjected to the CellTiter 96^®^ AQueous One Solution Cell Proliferation Assay (G3582; Promega Corp., Madison, WI, USA) and measured spectrophotometrically at 450 nm. The results were presented as the percentage of proliferation, where the proliferation of cells in culture medium without CCK-8 was set to 100%. Similar results were obtained in three independent experiments.

### Flow cytometry for cell cycle analysis

Cell cycle analysis was performed using flow cytometry following the harvesting and washing of the cells with cold PBS. Briefly, the cells were fixed in 75% ethanol and stored at −20°C for subsequent analysis. The fixed cells were subjected to centrifugation at 200 × g at 4°C for 5 min and washed twice with cold PBS. Ribonuclease A (final concentration, 20 μg/ml) and propidium iodide staining solution (final concentration, 50 μg/ml; Promega Corp.) were added to the cells, which were subsequently incubated for 30 min at 37°C in the dark. Cell analysis (≥100,000 cells) was performed using a FACSCalibur flow cytometry system (Becton Dickinson) equipped with CellQuest™ 3.3 software. ModFit™ LT 3.1 trial cell cycle analysis software (Verity Software House, Topsham, MA, USA) was used to determine the proportion of the cells in each of the different phases of the cell cycle.

### Tumor cell migration assays

The MCF-7 and HCC1937 cell migration assays were performed using a 24-well Transwell chamber. The MCF-7/HCC1937 cells from the four groups were separately resuspended in 100 μl medium without FBS and placed into the upper chamber of the 24-well Transwell culture system with 8.0-μm pore-size polycarbonate membrane inserts. The lower wells were filled with 600 μl medium supplemented with 20% FBS as a chemoattractant. Following 24 h of culture, the cells in the upper surfaces of the filter were removed with cotton swabs, and cells that had migrated to the lower surfaces were fixed with 4% paraformaldehyde, stained with 0.05% crystal violet and counted in five random fields on each filter. The membranes were dried thoroughly prior to examination. The number of cells that had migrated and adhered to the lower side of the membrane was assessed under high-power light microscopy (magnification, ×200). The average cell number (migrated cell number) was taken from five random fields per well and used to calculate the migration index: Migration index = migrated cell number of the experimental group/migrated cell number of the control group).

### Tumor cell invasion assays

The MCF-7 and HCC1937 cell invasion assays were performed using a 24-well Transwell chamber. The MCF-7/HCC1937 cells from the four groups were separately resuspended in 100 μl medium without FBS and placed into the upper chamber of a 24-well Transwell culture system with 8.0-μm pore-size polycarbonate membrane inserts coated with Matrigel™ for 1 h at 37°C. The lower wells were filled with 600 μl medium supplemented with 20% FBS as a chemoattractant. Following culture for 24 h, the cells on the upper surfaces of the filter were removed with cotton swabs, and the cells that had migrated to the lower surfaces were fixed with 4% paraformaldehyde, stained with 0.05% crystal violet and counted in five random fields on each filter. The membranes were dried thoroughly prior to examination. The cells that had migrated and adhered to the lower side of the membrane were counted under high-power light microscopy (magnification, ×200). The average cell number, taken from five random fields per well, was defined as the invaded cell number.

### hUMSCs/IL-18 migration assays

To assess the ability of the hUMSCs/IL-18 to migrate to the tumor cells, 5×10^4^ hUMSCs/IL-18 were dispersed onto the inserts of Transwell dishes (8-μm pore-size; Falcon™; Becton Dickinson Labware, Lincoln Park, MI, USA) and allowed to adhere for 1 h at 37°C. The Transwell inserts were then transferred to the bottom chamber, which contained 1×10^5^ tumor cells in 600 μl low-glucose DMEM supplemented with 10% FBS and cultured for 24 h. The cells on the upper surfaces of the filter were subsequently removed with cotton swabs, and any cells that had migrated to the lower surfaces were fixed with 95% alcohol, stained with 0.05% crystal violet and counted in five random fields on each filter.

### Statistical analysis

Statistical analyses were performed using a minimum of three independently prepared cultures and are presented as the mean ± standard deviation. Significant interactions were determined by either one-way or two-way analysis of variance and Bonferroni multiple comparison tests using Prism software (GraphPad Software, Inc., San Diego, CA, USA). A two-sided P<0.05 was considered statistically significant.

## Results

### Expression of IL-18 by hUMSCs

**Figure 1:**
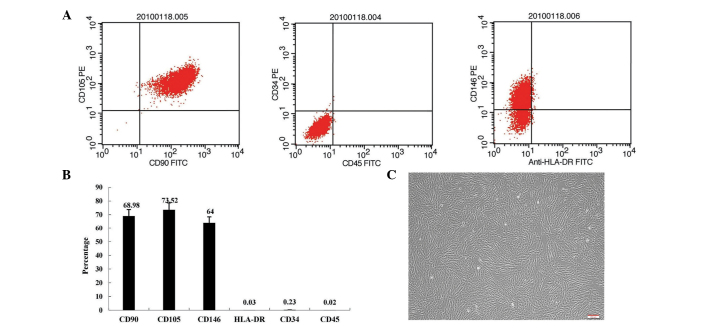
MSCs from human umbilical cord. Following the third passage, MSCs (A and B) exhibited CD146^+^, CD105^+^, CD90^+^, CD34^−^ and CD45^−^ expression, as determined using flow cytometry, and (C) were of high purity. MSC, mesenchymal stem cell; CD, cluster of differentiation; HLA, human leukocyte antigen; FITC, fluorescein isothiocyanate; PE, phycoerythrin. Magnification, ×100.

The hUMSCs that were expanded *in vitro* appeared similar to fibroblasts, with a characteristic spindle-shaped fusiform morphology (Fig. 1). Subsequent to the third passage, the cells were of high purity, with cluster of differentiation (CD)146^+^, CD105^+^, CD90^+^, CD34^−^ and CD45^−^ expression. No changes in cell shape were observed in the IL-18-transduced hUMSCs.

**Figure 2:**
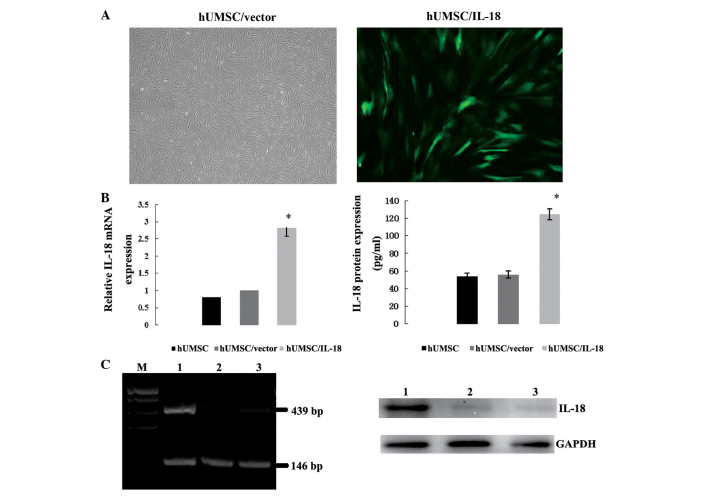
Expression of IL-18 protein by hUMSCs following transduction. (A) hUMSCs transfected with vector control and IL-18 gene by lentivirus (magnification, ×100). (B) Relative mRNA and protein expression of IL-18 in hUMSC/IL-18 cells, as determined by RT-PCR and western blotting, respectively. Relative mRNA expression of IL-18 in the hUMSCs/IL-18 group was higher compared with that in the hUMSCs/vector and hUMSCs groups, as determined by RT-PCR (^*^P<0.001). Protein expression of IL-18 in the hUMSCs/IL-18 group was higher compared with that in the hUMSCs/vector and hUMSCs groups (^*^P=0.007 vs. hUMSC/vector group, and P=0.008 vs. hUMSC group). (C) mRNA and protein expression of IL-18 in hUMSCs, as determined by RT-PCR and western blotting, respectively (lane 1, hUMSCs/IL-18; lane 2, hUMSCs/vector; lane 3, hUMSCs; M, marker). IL-18, interleukin-18; hUMSCs, human mesenchymal stem cells derived from umbilical cord; RT-PCR, reverse transcription-polymerase chain reaction.

GFP-containing lentivirus was utilized to assess transduction efficiency and the optimal viral infection conditions. Fluorescence microscopy revealed that the majority of the cell populations showed strongly positive GFP expression following transduction (Fig. 2A). Flow cytometric quantification of the GFP-positive cells showed a transduction efficiency of 85–92% at a multiplicity of infection (MOI) of 70; no significant benefit was obtained from increasing the MOI to 100.

To determine the expression of IL-18 in the hUMSCs, the cells and medium were collected and assessed using RT-PCR one week after transduction. RT-PCR showed that there was a 2.85±1.7-fold promotion of IL-18 expression in the hUMSCs/IL-18 group as compared with the hUMSCs/vector and hUMSCs groups (P<0.001, Fig. 2B). Protein expression was evaluated by western blotting and ELISA, which showed that the IL-18 concentration in the hUMSCs/IL-18 group was 125±16.7 pg/ml, as compared with 54±6.1 and 56±5.9 pg/ml in the hUMSCs/vector and hUMSCs groups, respectively (P=0.007 and 0.008, Fig. 2B).

### hUMSCs/IL-18 significantly suppress tumor cell growth in vitro

**Figure 3:**
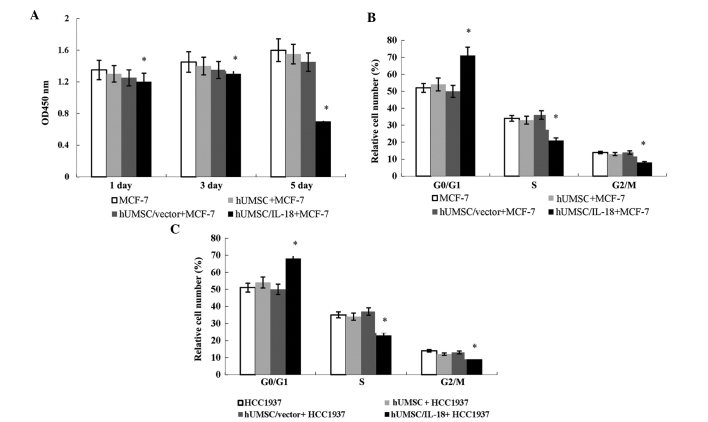
hUMSCs/IL-18 inhibit the proliferation of breast cancer cells. (A) Proliferation of MCF-7 cells was detected by cell counting kit-8 assay. A marked reduction in cell proliferation was observed in the MCF-7 cells following coculture with hUMSCs/IL-18, with an evident decrease in cell number compared with the other groups after a five-day culture period (^*^P<0.001). (B and C) Cell cycle analysis by flow cytometry in (B) MCF-7 and (C) HCC1937 cells (^*^P<0.05 vs. the other three groups). MCF-7 cells and HCC1937 cells cocultured with hUMSCs/IL-18 exhibited a significant increase in the percentage of cells in the G_1_ phase but decreased proportions in the S phase compared with the cells cocultured with hUMSCs/vector and hUMSCs (^*^P<0.05 vs. the other three groups). IL-18, interleukin-18; hUMSCs, human mesenchymal stem cells derived from umbilical cord; OD, optical density.

To evaluate the bioactivity of hUMSCs/IL-18 on cancer cell proliferation, the CCK-8 assay was performed in MCF-7 and HCC1937 cells. A marked reduction in cell proliferation was observed in the MCF-7 and HCC1937 cells following coculture with hUMSCs/IL-18, showing an evident decrease in cell number compared with the vector-control group after a five-day culture period (Fig. 3A).

To investigate the suppression mechanisms of hUMSCs/IL-18 on breast cancer cells, cell cycle analysis was performed. Flow cytometric analysis showed that hUMSCs/IL-18 significantly increased the percentage of cells in the G_0_/G_1_ phase but decreased that in the S and G_2_/M phase. As shown in Fig. 3B and C, the MCF-7 and HCC1937 cells cocultured with hUMSCs/IL-18 exhibited a significant increase in the percentages of cells in the G_1_ phase but a decreased proportion in the S phase compared with the cells cocultured with hUMSCs/vector and hUMSCs (P<0.05). This indicated that hUMSCs/IL-18 suppressed cancer cell proliferation by inducing the G_1_- to S-phase arrest of breast cancer cells.

### Effect of hUMSCs/IL-18 on the migration and invasion of tumor cells

**Figure 4:**
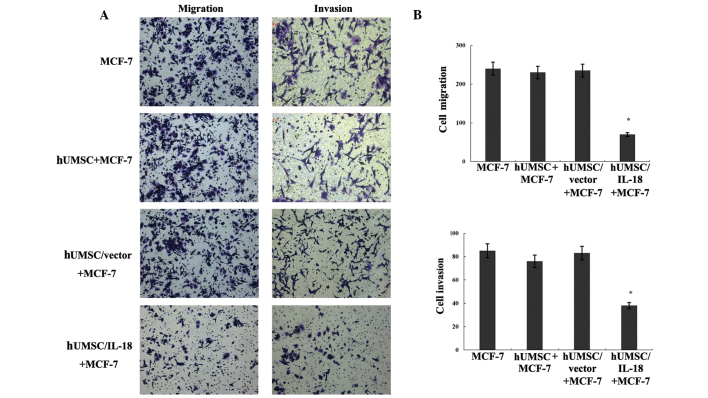
hUMSCs/IL-18 inhibit the migration and invasion of cancer cells. (A) Inhibition of migration and invasion of cancer cells in coculture with hUMSCs/IL-18, hUMSCs/vector or hUMSCs (stained with crystal violet). (B) Compared with hUMSCs/vector and hUMSCs, hUMSCs/IL-18 markedly suppressed the migration and invasion of the MCF-7 cells (^*^P<0.001). IL-18, interleukin-18; hUMSCs, human mesenchymal stem cells derived from umbilical cord. Magnification, ×200.

In order to investigate the effect of hUMSCs/IL-18 on the migration and invasion of MCF-7 and HCC1937 cells, cell migration and invasion assays were performed *in vitro* and the number of migrating and invading cells was counted. Compared with the hUMSCs/vector and hUMSCs groups, hUMSCs/IL-18 markedly suppressed the migration and invasion of the MCF-7 and HCC1937 cells (P<0.001), as shown in Fig. 4.

### hUMSCs/IL-18 migration towards tumor cells in vitro

**Figure 5:**
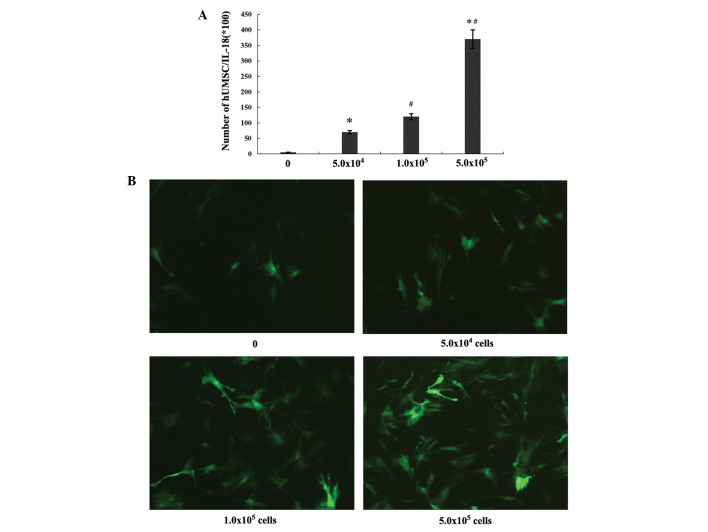
hUMSC migration towards tumor cells *in vitro*. (A) hUMSCs/IL-18 in the upper chamber were stimulated to migrate to the lower chamber by the addition of cancer cells into the lower chamber. The number of migrated hUMSCs increased in a dose-dependent manner with increasing numbers of cancer cells (^*^P=0.017, ^#^P=0.007 and ^*#^P=0.001 vs. no cancer cells in the lower chamber). (B) Fluorescent microscopic view of the migrated hUMSCs-green fluorescent protein in the lower chamber with different numbers of cancer cells (magnification, ×400). IL-18, interleukin-18; hUMSCs, human mesenchymal stem cells derived from umbilical cord.

The hUMSCs/IL-18 did not migrate to the lower chamber when the tumor cells were not present, but they were stimulated to migrate by the addition of tumor cells into the lower chamber. Of the hUMSCs/IL-18 (5.0×10^4^ cells) placed in the upper chamber, 14.0% (7×10^3^ cells) moved to the lower side in the presence of 5×10^4^ HCC1937 cells, 24% (1.2×10^4^ cells) moved to the lower side in the presence of 1.0×10^5^ HCC1937 cells and 74% (3.7×10^4^ cells) moved to the lower side in the presence of 5.0×10^5^ HCC1937 cells. The migration of the hUMSCs increased in a dose-dependent manner with increasing numbers of tumor cells (P<0.001, Fig. 5).

## Discussion

In the present study, hUMSCs genetically modified with IL-18 gene were used to study the effect of hUMSCs/IL-18 on the growth, migration and invasion of two breast cancer cell lines *in vitro*. Through the use of a lentivirus, IL-18 protein was successfully secreted from the hUMSCs/IL-18 at an MOI of 70. Furthermore, the results showed that hUMSCs/IL-18, but not hUMSCs, significantly inhibited the growth, migration and invasion of MCF-7 and HCC1937 cells *in vitro*.

The present study showed that hUMSCs could be efficiently modified by lentiviral systems and could stably express the transgene. In a previous study by Brennen *et al* (23), it was shown that MSCs engrafted in tumors could act as stromal precursor cells and successfully function as cellular vehicles for gene delivery and contribute to the local production of biological agents. Compared with other types of cells, MSCs are simpler to obtain and can be more easily propagated *in vitro*; furthermore, their use is associated with fewer ethical issues and immune response problems. hUMSCs possess specific chemoresistance and migration properties and should therefore be considered as a valuable type of adult stem cell for use in cancer therapy (1,24). In the present study, the GFP and IL-18 genes did not show any cytotoxic effects on the transduced hUMSCs, and a high active expression of IL-18 was observed. A possible risk associated with lentiviral transduction is insertional oncogenesis following multiple vector integrations into the host genome. Cell-based therapy with systemic delivery of MSCs has been used in animal experiments and clinical trials, and no adverse effects have been reported to date (11,25).

MCF-7 and HCC1937 cells are human breast cancer cell lines and respectively represent luminal and triple-negative breast cancer cells. The migration of hUMSCs towards the two cancer cell lines was demonstrated *in vitro* in the present study. This observation was consistent with previous reports (26,27) and represents an important characteristic when enforced *in vivo*; however, the mechanisms underlying the homing and engrafting of the hUMSCs to the tumors have yet to be fully elucidated. It is likely that hUMSCs express a wide range of cytokine and chemokine receptors, and that differential gene regulation may occur following the exposure of the hUMSCs to different microenvironments. Since tumor cells and their microenvironments secrete chemokines or cytokines, these may be responsible for upregulating the expression of the chemokine and cytokine receptors on the hUMSCs (28,29), such as C-X-C chemokine receptor type 4 (which is upregulated in hUMSCs) and stromal cell-derived factor 1 (secreted by hUMSCs), leading to an effect on MSC migration (30,31).

The antitumor activities of hUMSCs/IL-18, i.e. the inhibition of tumor cell growth, as well as migration and invasion, were confirmed by coculture in Transwell chambers in the present study. Compared with hUMSCs and hUMSCs/vector, hUMSCs/IL-18 could significantly suppress the proliferation, migration and invasion of the MCF-7 and HCC1937 cells. The mechanisms by which hUMSCs/IL-18 caused the growth attenuation of cancer cells were determined by cell cycle analysis with flow cytometry. The results indicated that the hUMSC/IL-18-dependent tumor cell growth attenuation occurred predominantly due to cell cycle arrest at the G_1_/S check point.

IL-18 has been demonstrated to promote the production of Th1-type cytokines, which are involved in the antitumor cytotoxic T-cell response (22). Consistent with the theory that Th1 cells are associated with immunity to cancer, the administration of IL-18 can result in notable antitumor effects. IL-18 has been found to induce IFN-γ and granulocyte-macrophage colony stimulaing factor secretion by T and NK cells, enhance the cytolytic activity of NK cells, increase the proliferation of T cells and activate CD8^+^ cytotoxic T lymphocytes (32–34). IL-18 can also induce IL-2 secretion by Th1 clones, stimulated by immobilized anti-CD3, and enhance NK cell expression of the DC-attracting chemokines C-C motif chemokine 3 (CCL3), CCL4, Chemokine (C-X-C motif) ligand 8 and XC chemokine ligand 1, resulting in the attraction of immature DCs. Previous studies have demonstrated that DCs loaded with lysed tumor cells and IL-18 may induce Th1 responses against glioma antigens (19,22). Such IL-18-driven enhancement in the expression of DC-attracting chemokines corresponds closely to the previously reported regulation of IFN-γ and tumor necrosis factor-α (TNF-α), factors essential for the NK cell-mediated activation of DCs, in IL-18-primed human NK cells (34). These results indicate that IL-18 can be used in cancer immunotherapy as a promoter of a strong cellular response.

Although hUMSCs/IL-18 significantly suppressed the growth and invasion of cancer cells, IL-18 has also been found to promote tumor progression. Increased levels of IL-18 have been detected in certain types of cancer, and elevated IL-18 expression has been associated with tumor growth and metastasis in breast cancer (20). In the present study, a novel possibility that IL-18 could have a function in the treatment of breast cancer was revealed; however, further experiments are necessary to verify the effect of hUMSCs/IL-18 on breast cancer *in vivo*. Furthermore, combinations with other cytokines, such as IL-12 and TNF-α, or with a DNA vaccine, could enhance the antitumor efficacy in the future.

In conclusion, the present study has demonstrated the therapeutic potential of hUMSCs/IL-18 in breast cancer treatment. It was demonstrated that genetically engineering hUMSCs to produce IL-18 had synergistic therapeutic benefits and that such cells could contribute to an adoptive immunotherapy for breast cancer and thereby provide a promising new treatment option. Thus, further investigation into the use of hUMSCs/IL-18 as vehicles of tumor therapy *in vivo*, including pilot clinical trials, and in combination with other therapies, such as surgery, chemotherapy, endocrine therapy and targeted therapy, is warranted.
