# [Corrigendum] Mesenchymal stem cells expressing interleukin‑18 suppress breast cancer cells *in vitro*

**DOI:** 10.3892/etm.2023.12131

**Published:** 2023-07-24

**Authors:** Xiaoyi Liu, Jianxia Hu, Suyuan Sun, Funian Li, Weihong Cao, Yu Wang, Zhongliang Ma, Zhigang Yu

Exp Ther Med 9:1192-1200, 2015; DOI: 10.3892/etm.2015.2286

Subsequently to the publication of the above article, a concerned reader drew to the authors’ attention that, with the invasion and migration assay data shown in Fig. 4A on p. 1197, there were two pairs of apparently overlapping data panels featured in this figure (specifically, the ‘MCF-7/Invasion’ and ‘huMSC+MCF-7/Invasion’ data panels, and the ‘huMSC+MCF-7/Migration’ and ‘huMSC/Vector/Migration’ data panels).

After having consulted their original data, the authors have realized where the errors occurred during the process of assembling this figure. The revised version of Fig. 4, now showing the correct data for the ‘huMSC+MCF-7/Invasion’ experiment and new data from one set of replicate experiments for all the migration assay data (shown in the left column of panels in the figure), is featured on the next page. Note that these errors did not grossly affect either the results of the conclusions reported in this study, and all the authors agree with the publication of this corrigendum; furthermore, they apologize to the Editor of *Experimental and Therapeutic Medicine* and to the readership for any inconvenience caused.

## Figures and Tables

**Figure 4:**
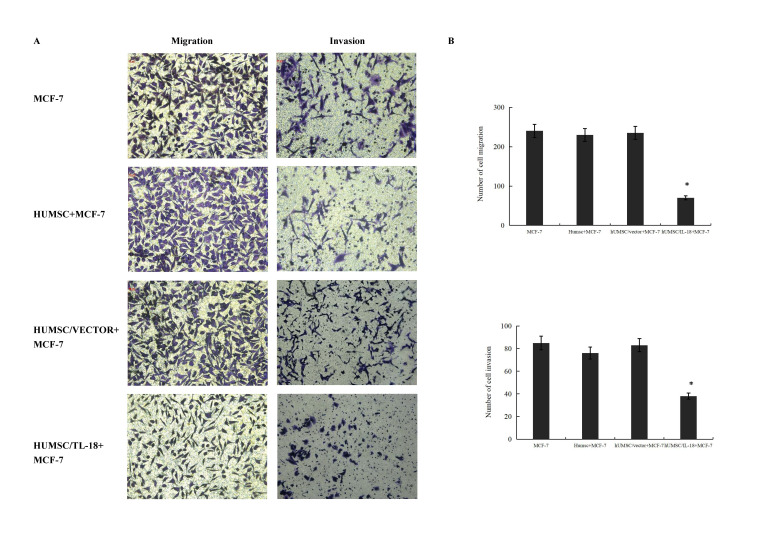
hUMSCs/IL-18 inhibit the migration and invasion of cancer cells. (A) Inhibition of migration and invasion of cancer cells in coculture with hUMSCs/IL-18, hUMSCs/vector or hUMSCs (stained with crystal violet). (B) Compared with hUMSCs/vector and hUMSCs, hUMSCs/IL-18 markedly suppressed the migration and invasion of the MCF-7 cells (^*^P<0.001). IL-18, interleukin-18; hUMSCs, human mesenchymal stem cells derived from umbilical cord. Magnification, ×200..

